# ATP Synthase: The Right Size Base Model for Nanomotors in Nanomedicine

**DOI:** 10.1155/2014/567398

**Published:** 2014-01-29

**Authors:** Zulfiqar Ahmad, James L. Cox

**Affiliations:** Department of Biochemistry, Kirksville College of Osteopathic Medicine, A. T. Still University of Health Sciences, Kirksville, MO 63501, USA

## Abstract

Nanomedicine results from nanotechnology where molecular scale minute precise nanomotors can be used to treat disease conditions. Many such biological nanomotors are found and operate in living systems which could be used for therapeutic purposes. The question is how to build nanomachines that are compatible with living systems and can safely operate inside the body? Here we propose that it is of paramount importance to have a workable base model for the development of nanomotors in nanomedicine usage. The base model must placate not only the basic requirements of size, number, and speed but also must have the provisions of molecular modulations. Universal occurrence and catalytic site molecular modulation capabilities are of vital importance for being a perfect base model. In this review we will provide a detailed discussion on ATP synthase as one of the most suitable base models in the development of nanomotors. We will also describe how the capabilities of molecular modulation can improve catalytic and motor function of the enzyme to generate a catalytically improved and controllable ATP synthase which in turn will help in building a superior nanomotor. For comparison, several other biological nanomotors will be described as well as their applications for nanotechnology.

## 1. **Introduction**

Biological motors are molecular machines found in living systems. These nanomachines are designed to carry out specific functions. In order to perform their designated jobs they use energy and convert it to mechanical work. The majority of protein based molecular nanomotors use chemical energy ATP to perform mechanical work [1]. Molecular size nanomotors are commonly divided into two categories: (I) biological and (II) nonbiological. In this review we will focus on biological nanomotors, particularly ATP synthase. Biological nanomotors are incredible molecular machines which drive fundamental processes of life. In addition to F_1_F_0_ ATP synthase bacterial flagella, kinesin, dynein, myosin, actin, microtubule, dynamin, RNA polymerase, DNA polymerase, helicases, topoisomerases, and viral DNA packaging motors are some other prominent biological nanomotors.

In recent years many laboratories [2–10] have been trying to create synthetic or nonbiological nanomotors, which is not the topic of this review. However, before discussing the biological nanomotors it would be helpful to briefly go over nonbiological nanomotors too. The purpose of creating nonbiological nanomotors by mimicking the biological nanomotors is to get the desired physiological function done within the living systems. Interestingly, the nonnatural nanodevices generally happen to be less efficient compared to their biological counterparts. Scientists in the field of nanotechnology are continuously reconnoitering the possibility of creating molecular motors *de novo*. These synthetic molecular motors currently suffer many limitations that confine their use to the research laboratory only. However, many of these deficiencies can easily be dealt with comprehensive knowledge of known biological nanomotors. Thus, the answer to the valid question of how to manage the functional capabilities of the nanomotors which could be used inside the living system can be found in the naturally occurring biological nanomotors. In this review we advocate that it is of paramount importance to have a base model in order to develop nanomotors for nanomedicine usage. We also suggest that ATP synthase best exemplifies the nanomotor for a right size base model.

## 2. F_1_F_0_ ATP Synthase 

Among all known biological nanomotors, ATP synthase stands alone for being the universal nanomotor found in all living systems from bacteria to man. Ability of molecular modifications of ATP synthase catalytic sites is of additional advantage. In order to synthesize ATP, the cell's energy currency, ATP synthase uses a mechanical rotation mechanism to utilize the energy generated by oxidation of foodstuffs. ATP synthase enzyme is critical to human health and is likely to contribute to new therapies for multiple diseases, such as cancer, bacterial infections, and obesity, that affect both people and animals [11–15].

### 2.1. General Features

**Figure 1:**
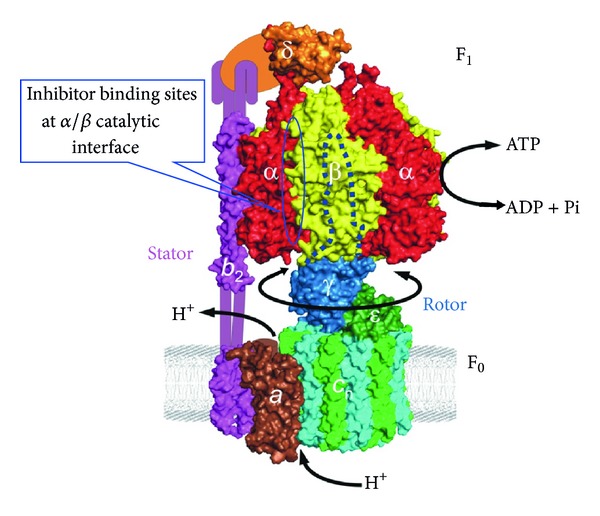
*Escherichia coli*  F_1_F_0_ ATP synthase structure: *E. coli* ATP synthase in the simplest form contains water soluble F_1_ and membrane bound F_0_ sectors. Catalytic activity ensues at the *α*/*β* interface of F_1_ sector. Many inhibitors also bind to the F_1_ sector which comprises five subunits (*α*_3_ *β*_3_ *γδε*). The proton pumping occurs at the F_0_ sector comprising three subunits (ab_2_c). This structure of *E. coli*  F_1_F_0_ ATP synthase is reproduced from Weber [27] with permission; copyright Elsevier.

Figure 1 shows the general structural and functional aspects of ATP synthase in its simplest form found in *Escherichia coli* with a total molecular size of ~530 kDa and contains eight different subunits, namely, *α*_3_ *β*_3_ *γδε*ab_2_c_10–15_. F_1_ corresponds to *α*_3_ *β*_3_ *γδε* and F_0_ to ab_2_c_10–15_. In chloroplast and mitochondria the general structure is similar to *E. coli* except that there are two isoforms and 7–9 additional subunits, respectively. It is also known that as a complex they contribute only to a small fraction of additional mass and may have regulatory roles [16–18]. F_1_F_0_-ATP synthase is the smallest known biological nanomotor, found in almost all living organisms including plants, animals, and bacteria. This enzyme is responsible for ATP synthesis by oxidative or photophosphorylation in membranes of bacteria, mitochondria, and chloroplasts. Thus, ATP synthase is the central means of cell energy production in animals, plants, and almost all microorganisms. A typical 70 kg human with a relatively sedentary lifestyle will generate around 2.0 million kg of ATP from ADP and Pi (inorganic phosphate) in a 75-year lifespan. Present understanding of the F_1_F_0_ structure and mechanism can be found in references [4, 11, 14, 19–41].

ATP hydrolysis and synthesis occur on three catalytic sites at the interface of *α*/*β* subunit in the F_1_ sector, whereas proton transport occurs through the membrane embedded F_0_ sector. Proton gradient-driven clockwise rotation of *γ* (as viewed from the membrane) leads to ATP synthesis and anticlockwise rotation of *γ* results in ATP hydrolysis [15]. The *γε*c_*n*_ forms the part of rotor, while b_2_ *δ* is the part of stator in ATP synthase [38, 42–44].

The production of ATP reaction in the three catalytic sites ensues sequentially. In this reaction mechanism, the three catalytic sites have altered affinities for nucleotides at any moment, and each undergoes conformational transitions which results in the direction of substrate (ADP + Pi) binding→ATP synthesis→ATP release. In other words catalysis requires sequential involvement of three catalytic sites where each catalytic site changes its binding affinity for substrates and products as it proceeds through the cyclical mechanism known as “binding change mechanism” initially proposed by Boyer [45–51].

Proton motive force is converted in F_0_ to mechanical rotation of the rotor shaft, which drives conformational changes of the catalytic domains in F_1_ to synthesize ATP. Conversely, hydrolysis of ATP induces reverse conformational changes and consequently reverses rotation of the shaft. Rotation of *γ* subunit in isolated *α*_3_ *β*_3_ *γ* subcomplex has been observed directly by Yoshida, Kinosita, and colleagues in Japan and subsequently by several other labs [4, 25, 36, 52–58]. The reaction mechanism of ATP hydrolysis and synthesis in F_1_F_0_ and their relationship to the *γ*-subunit mechanical rotation is a fundamental question, has relevance to nanotechnology, and applies to many ATPases and GTPases [59, 60].

### 2.2. Nature and Modulation of the Catalytic Site Residues

Experimental data shows that modulations of catalytic sites can result in enhanced catalytic and motor functions of ATP synthase [61, 62]. For the purpose of catalytic site modifications of ATP synthase it is important to understand the terms associated with catalytic sites and the residues involved in it. According to X-ray crystallographers the three catalytic sites of ATP synthase are *β*TP for ATP, *β*DP for ADP, and *β*E the empty state to which Pi (inorganic phosphate) binds [63–65]. Also, in active cells, the cytoplasmic concentrations of ATP and Pi are approximately in the 2–5 mM range, whereas that of ADP is at least 10–50-fold lower. Equilibrium binding assays have established that both ADP and ATP bind to catalytic sites with relatively similar binding affinities [66–69]. During ATP synthesis, proton gradient-driven rotation of subunits drives *β*E the empty catalytic site to bind Pi tightly, thus stereochemically precluding ATP binding and therefore selectively favoring ADP binding [28].

**Figure 2:**
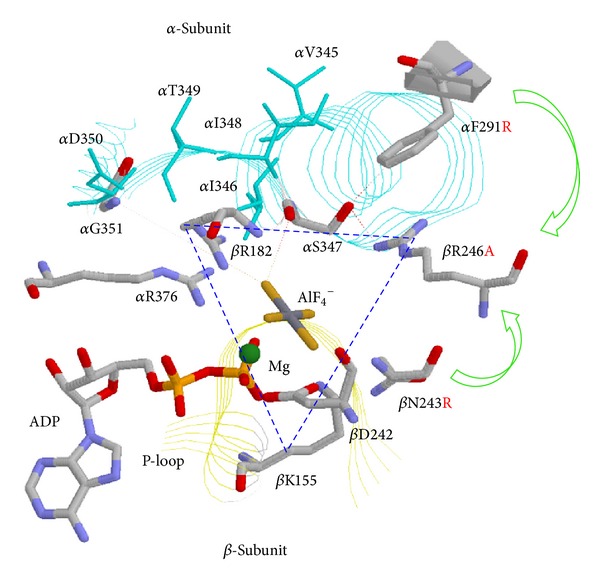
Catalytic sites X-ray structure of ATP synthase depicting spatial relationship between *α* and *β*-subunit residues. The *β*DP site in the AlF_4_^−^-inhibited enzyme structure is taken from [63]. *E. coli* residue numbering is used. It can be seen that removal of arginine from *β*R246 can be compensated by introduction of arginine in the neighboring residues *α*F291 or *β*N243. Dotted triangle shows the residues *β*Lys-155, *β*Arg-182, *β*Arg-246, *α*Arg-376, and *α*Ser-347, forming a triangular Pi binding site. Figure was modified from the originally published figure in [75]. RasMol molecular visualization software was used to generate the figure.

Physical and chemical properties of amino acids in general and positive charges in particular play critical role in the catalytic sites. Positively charged arginine residues are known to occur with high propensity in Pi binding sites of proteins generally and in the Pi binding site of *β*E catalytic site of ATP synthase specifically [70]. Earlier [22, 33, 61, 62, 71–75] it was found that removal of positive charge from the catalytic site Pi binding subdomain abrogates Pi binding and Pi binding was restored by replacing a nonpositive neighboring residue with positively charged arginine (see Figure 2). This study signifies the importance of modulation of charge in the phosphate binding site of *Escherichia coli* ATP synthase. It was found that by inserting positive charges in incremental order in specific positions in the catalytic site, it is possible not only to restore Pi binding but also to enhance the catalytic activity of the enzyme. This possibility of catalytic modification is of high value in the creation of a catalytically controllable, superior biological nanomotor.

### 2.3. Role in Disease Conditions

Normal functioning of ATP synthase is indispensable to human health. Although failure of the ATP synthase complex is implicated in wide variety of diseases but simultaneously this enzyme may also be used as a therapeutic drug target in the treatment of many disease conditions such as cancer, tuberculosis, obesity, Alzheimer's, and microbial infections [14, 15, 21, 76, 77]. Subunit malfunctions in ATP synthase are the cause of many diseases; for example, the c-subunit of ATP synthase is involved in neuro degenerative Battens disease [78]. Buildup of *α*-subunit and reduction of *β*-subunit in the cytosol is seen in Alzheimer's disease patients [79, 80]. Subunit F6 has been associated with hypertension [81, 82]. Occurrence of ATP synthase on the multiple animal cell surfaces is linked with many cellular processes, for example, angiogenesis, intracellular pH regulation, and programmed cell death [83–91]. Moreover, the inhibition of nonmitochondrial ATP synthase was found to cause the inhibition of cytosolic lipid droplet buildup making ATP synthase an appropriate molecular target for antiobesity drugs [92].

### 2.4. Potential Molecular Drug Target

So far approximately twelve binding sites for a variety of natural and synthetic inhibitors have been identified on ATP synthase. Thus the use of ATP synthase as a potential molecular drug target seems straight forward. Recently the role of dietary polyphenols and peptides as antimicrobial and antitumor molecules targeting ATP synthase came to prominence [15, 21, 93–97]. Defense against dental cavities caused by the microbe *Streptococcus mutans* presents an amazing example for this potential. Inhibition of *S. mutans* ATP synthase provides a prophylactic effect against *S. mutans* metabolism by arresting biofilm formation and acid production [98, 99]. Another valuable example comes from *Mycobacterium tuberculosis* ATP synthase, where two mutations (D32V and A63P) in its c-subunit cause resistance to the tuberculosis drug diarylquinoline [100, 101]. The importance of ATP synthase as a promising target for drug development is also evident from the fact that many antibiotics such as efrapeptins, aurovertins, and oligomycins inhibit its function [21, 102–106].

### 2.5. ATP Synthase as a Nanomotor

It's noteworthy that ATP synthase has been widely studied by many laboratories using biochemical, biophysical, genetic, and molecular biology techniques. X-ray crystallographic studies have clarified the detailed subunit composition. The coupling between the mechanical force generated by rotation of subunits and chemical reactions of ATP synthase has also been elucidated [107–109]. The smallest known single molecule of F_1_-ATPase acting as a rotary motor by direct observation of its motion was observed first time in 1997 by Noji et al. [36]. They suggested that the *γ*-subunit of F_1_-sector rotates within the *α*/*β* interface. This speculation has been supported by structural, biochemical, and spectroscopic studies. They attached a fluorescent actin filament to the *γ*-subunit and detected the motion directly. In the presence of ATP, the F_1_ rotated for more than 100 revolutions. The single-molecule measurements of rotation catalyzed by the F_1_F_0_ ATP synthase from Frasch lab [4] have provided new insights into the molecular mechanisms of the F_1_F_0_ molecular nanomotors. Finally universal presence of ATP synthase makes it more fascinating than other known biological nanomotors which are restricted to certain species, cells, or tissues. This enzyme can also work both in forward and reverse directions.

## 3. Bacterial Flagella

### 3.1. General Features

Flagellum is an attachment that overhangs from the body of some eukaryotic and prokaryotic cells. The established role of the flagellum is propulsion but it is also sensitive to chemicals and temperatures outside the cell, thus functioning as a sensory organelle. However, while both prokaryotic and eukaryotic flagella are used for swimming, they vary significantly in protein composition, structure, and mechanism of propulsion [110, 111]. The bacterial flagellum is made up of the protein flagellin and is driven by a protein rotary engine (the Mot complex), located at the flagellum's anchor point on the inner cell membrane. The flagellum is powered by a proton motive force. H^+^ ions move across the cell due to a concentration gradient. Some bacterial species flagella are driven by a sodium ion pump rather than a proton pump [112].

### 3.2. Rotatory Properties

The rotary action transports protons across the membrane. Although the rotor part itself can operate at ~6–15 K rpm, flagella filament typically attain a maximum speed of 200–1000 rpm. The tubular shape of flagella is suited to movement of microscopic organisms, where the viscosity of the surrounding water is much more important than its mass or inertia [113]. The intensity of proton motive force controls the rotational speed of flagella which in turn permits extraordinary speed in proportion to their size. Some bacteria can achieve up to 60 times to their cell length per second [114–116].

### 3.3. Structural and Functional Properties

Structurally bacterial flagellar nanomotors consist of a nonrotating stator part composed of MotA and MotB proteins and rotor made of FliG, FliM, and FliN proteins. The stator complex couples ion flow to rotation through cyclical conformational changes in MotB protein. The rotor complex is also referred to as “switch complex” because it can mediate counterclockwise to clockwise (CCW to CW) reversals. The switch complex forms a large cylindrical ring (C-ring) comprised of multimeric rings of FliG, FliM, and FliN proteins. Chemotactic protein phospho-CheY binds to FliM to signal a direction switch through FliG. A CCW to CW switch occurs with a conformational change in FliG subunit [117]. Interestingly, a three-amino acid deletion mutant of FliG has been studied which is locked in the CW direction [118].

### 3.4. The Base Model Question

The question of paramount importance is whether or not bacterial flagella can be used as a base model in the development of nanomotors in nanomedicine usage. Since it would be difficult to reconstitute flagellar motors from isolated motor proteins, most work in this area employs intact cells with preassembled motors. The attendant problem of limited cell lifetime could be best overcome if “old” cells could be swapped out with new cells. One application for nanotechnology of the flagellar nanomotor is as a living fluid mixer [119]. A tethered flagellum allows rotation of *E. coli* cells at about 240 rpm to drive local solution mixing. Construction of a hybrid microrotary motor driven by *Mycoplasma mobile* cells was achieved by Hiratsuka et al. [120]. Although gliding bacteria differ in mechanism from bacteria flagellar motors, mechanical walking of rod-like structures driven by motors is believed to be involved. In these studies continuous rotation of 20 *μ*m SiO_2_ fabricated rotors at about 2 rpm was observed. This was the first example of “flagellar motors” driving microfabricated structures. Microdevices which employ bacterial flagellar motors for fluid transport or mixing have been fabricated [121]. Patterning of attached bacteria in these microdevices showed linear velocities of microspheres up to 150 *μ*ms^−1^. Motile bacteria which exhibit magnetotaxis, such as strain MC-1, a marine coccus, are being developed as drug targeting vehicles [122]. Advantages for these motile organisms are *in vivo* steerability and external control by MRI systems favors this type of “nanorobot”. Technical hurdles, such as bacterial navigation in large blood vessels, still need to be overcome. MRI imaging of these motile bacteria truly sets this system apart and bodes well for near future applications in medicine.

## 4. Kinesins

### 4.1. General Features

A kinesin is another motor protein found in eukaryotic cells. Kinesins are ATPases which require ATP hydrolysis for their movement along the microtubule filaments. Several cellular tasks such as mitosis, meiosis, and transport of cellular cargo, for example, axonal transport are achieved by active movement of kinesins. The kinesins are responsible for anterograde or outward transport of cargo from the cell center. Primarily, kinesins were discovered as microtubule based anterograde intracellular transport nanomotors [123]. In recent years as the kinesin superfamily became very large a variety of naming patterns started floating around, leading to duplication and confusions. To address this and other issues that were in the classification, American Society for Cell Biology meeting in 2003 formulated a standardized kinesin nomenclature based on 14-family designations [124–126].

### 4.2. Overall Structure

The overall structure of kinesin superfamily members differs but the exemplary kinesin-1 consists of heavy (KHCs) and light chains (KLCs), thus forming a heterotetramer. The motor domain or the globular head of the KHC at the amino terminal end is connected to the stalk, a long alpha-helical coiled-coil domain, which ends in a carboxy terminal tail domain and in turn is associated with the light-chains. The stalks of two KHCs intertwine to form a coiled-coil that directs dimerization of the two KHCs. Mostly transported cargo binds to the KLC but in some cases cargo can also bind to the KHC c-terminal domains [127].

### 4.3. Motor Domain

Amino acid sequence of the globular head domain is highly conserved among various kinesins. The globular head has two discrete binding sites for the microtubule and for ATP. ATP binding → ATP hydrolysis → ADP release causes the conformational changes in the microtubule-binding domains that result in the movement of the kinesin. Kinesins are structurally related to G proteins, which hydrolyze GTP instead of ATP. Nanomotor proteins such as kinesins transport large cargo by unidirectional walking along the microtubule tracks by hydrolyzing one ATP molecule at each step [128]. Multiple kinesin nanomotors are also known to cooperatively transport the cargoes *in-vivo *[129–132]. The detailed discussion on ATP powered step-wise movement of kinesin head along with microtubules can be found in the references [133, 134].

### 4.4. Applicability in Nanomedicine

Applications of kinesin nanomotors to nanotechnology continue to evolve. Two basic configurations for kinesin motors are fixing of kinesins to fluidic channels to propel microtubules or microtubule immobilization for kinesin tracking. Steering of microtubules has been achieved by application of an electric field perpendicular to kinesin coated microfluidic channels [135]. By reversing the electric field, sorting of red or green labeled microtubules to left and right collecting reservoirs was achieved. One goal for kinesin nanomotors is the development of microfluidic devices which can deliver specific analytes from cargo loading chambers to detector ports, thus achieving sorting and concentration functions. A major drawback for the use of kinesins for nanotechnology is a deficiency of specific docking systems of the cargo.

## 5. Dyneins 

Like kinesins dyneins are also cytoskeletal type of molecular motor proteins, which require the energy from ATP to perform mechanical work. In contrast to the kinesins, which transport the cellular cargo from the center of the cell towards the periphery, the plus-end dyneins transport cellular cargo towards the cell center, the minus-end of the microtubule. Thus dyneins and kinesins are named minus-end and plus-end directed nanomotors, respectively.

Dynein walks in such a way that at any given time one of its stalks is continuously attached to the microtubule. This allows the dynein to move a substantial distance along the microtubule without detaching. Cytoplasmic dynein helps transport cargo needed for cell functions and is also involved in the movement of chromosomes and positioning the mitotic spindles for cell divisions [136]. Dynein as a motor is a complex protein assembly composed of many smaller polypeptide subunits.

Dynein has a molecular mass of about 1.5 MDa and comprises nearly twelve polypeptide subunits. Two of them are identical heavy chains of ~520 kDa containing the ATPase activity and are thus responsible for generating movement along the microtubule. Two are 74 kDa intermediate chains which are thought to attach the dynein to its cargo; four are ~56 kDa intermediate chains; and the rest are less known light chains. Also, another multisubunit protein dynactin (dynein activator complex), essential for mitosis, regulates the activity of dynein. Dynein gets activated by binding to dynactin which in turn facilitates cargo attachment to dynein [137]. Due to the more complex structure of dyneins compared to the kinesins, applications to nanotechnology have yet to be developed.

## 6. Myosin

Myosin is a family of ATP-dependent motor proteins and is mainly involved in muscle contraction. Structurally myosin molecule is composed of two large polypeptide heavy chains and four smaller light chains. Both heavy and light chain polypeptides combine to form two globular heads, while only heavy chains intertwine to form the tail part. The myosin molecules makeup the core of thick filament and remain oriented in opposite directions. Each globular head contains two binding sites one for actin and other for ATP. Overall muscle contraction requires involvement of another thin filament protein actin. In essence myosin provides actin-based motility [138–140].

As far as myosin's role in nanomedicine is concerned investigations into use of contractile cell grafts for myocardial regeneration have begun [141]. Also, the transport of liposome tethered to bundled actin over myosin coated surfaces has also been examined [142]. The applications of myosin-actin in nanomedicine are still in infancy stages and are just emerging.

While detailed structural and functional aspects of F_1_F_0_ ATP synthase are available to use for its role in nanomedicine for other nanomotors it still seems a long way to go. Analysis of all biological nanomotors shows that many nanomotor proteins link catalytic ATP utilization to linear, unidirectional force generation. More is known of the kinesins and myosins than dyneins, primarily due to the greater molecular complexity of the latter type. Kinesins are microtubule motors which consist of 45 members in the mammalian kinesin superfamily. Kinesins are involved in cargo transport in cells by tethering different cargo vesicles to divergent tail domains. Most kinesins track processively along microtubules towards the plus end with a few members of the family tracking to the minus end [143]. Force production of kinesins, and structurally related myosins, involves conformational changes in motor proteins converting strain relieving recoil into force generation [144]. A kinesin force-producing conformational change within the motor protein, upon ATP binding, results in altered motor to filament interaction. Processive movement of the kinesin is believed to be due to chemical gating which requires communication between the two motor headpieces. Conserved motifs within the kinesins are the nucleotide binding P-loop and switch I and II loops [145]. During the nucleotide catalytic cycle small movements of the loop regions are transmitted to movement of the neck linker segment.

In this review we have focused on nanomotors that have shown some promise of being applicable in nanomedicine. Many biological nanomotors such as myosin, actin, microtubule, dynamin, RNA polymerase, DNA polymerase, helicases, topoisomerases, and viral DNA packaging motors currently have few nanomedicine applications; therefore they are subject of a separate future review article.

## 7. Conclusions

Being relatively new nanobiology or nanobiotechnology covers a variety of related technologies. This is basically a merger of molecular biology with technology that covers nanodevices, nanoparticles, protein motors, and other nanoscale phenomenon in the living cells. One of the objectives behind nanobiology is to apply nanotools to solve relevant medical/biological problems. Developing new tools and refining them for delivering better health care is another principal objective of nanotechnology [146].

Overall rotatory motor functions and universal presence makes F_1_F_0_ ATP synthase a front runner base model in the development of nanomotors for nanomedicine usage. Moreover, the first tentative steps in nanotechnology with biological nanomotors have begun. It will be of great interest to see the development of hybrid technologies which link microfabrication to various biological nanomotors. The realization of the full potential in this exciting area will begin to occur when useful devices driven by nanomotors appear.
